# Pharmacokinetics and Pharmacogenomics of Ribociclib in Black Patients with Metastatic Breast Cancer: The LEANORA study

**DOI:** 10.21203/rs.3.rs-4656461/v1

**Published:** 2024-08-13

**Authors:** Sandra Swain, Ilana Schlam*, D. Max Smith*, Cody Peer, Tristan Sissung, Keith Schmidt, Ming Tan, Ami Chitalia, Nanette Bishopric, Seth Steinberg, Hyoyoung Choo-Wosoba, Giulia Napoli, Christopher Gallagher, Nadia Ashai, Kristen Whitaker, Candace Mainor, Shruti Tiwari, Nicole Swanson, Stacy Malloy, Claudine Isaacs, William Figg

## Abstract

Underrepresented populations' participation in clinical trials remains limited, and the potential impact of genomic variants on drug metabolism remains elusive. This study aimed to assess the pharmacokinetics (PK) and pharmacogenomics (PGx) of ribociclib in self-identified Black women with hormone receptor-positive (HR+)/human epidermal growth factor receptor 2-negative (HER2) advanced breast cancer. LEANORA (NCT04657679) was a prospective, observational, multicenter cohort study involving 14 Black women. PK and PGx were evaluated using tandem mass spectrometry and PharmacoScan™ microarray (including CYP3A5*3 , *6 , and *7 ). CYP3A5 phenotypes varied among participants: 7 poor metabolizers (PM), 6 intermediate metabolizers (IM), and one normal metabolizer (NM). The area-under-the-curve did not significantly differ between PMs (39,230 hr*ng/mL) and IM/NMs (43,546 hr*ng/mL; p = 0.38). The incidence of adverse events (AEs) was also similar. We found no association between *CYP3A5* genotype and ribociclib exposure. Continued efforts are needed to include diverse populations in clinical trials to ensure equitable treatment outcomes.

**Additional Declarations:** There is a conflict of interest DMS reports research funding to institution from Kailos Genetics, Inc. MTT reports receiving honoria from AstraZeneca, Incyce, Otsuka, Sanofi Pasteur, consulting for American Gene Technologies, and receiving research funding to institution from Genentech. NHB reports they or an immediate family member have consulted for Catena, a leadership role for Seagen, stock or ownership in Seagen, Lilly, Gilead Sciences, and Pfizer. CG reports consulting for Daiichi Sankyo, Illy, Biotheranostics, and Pfizer and Speakers' Bureau for Daiichi Sankyo/UCB Japan. KDW reports consults for Biotheranostics and receiving honoraria from MHJ Life Sciences. CBM reports receiving research funding to institution from Pfizer and Cantex Pharmaceuticals. CI reports consulting for Pfizer, Novartis, Puma Biotechnology, Seagen, Ion Solutions, AstraZeneca/MedImmune, Gilead Sciences, receiving travel support from Pfizer, holding patents, royalties, or other intellectual property from McGraw Hill Publishing, UpToDate (Wolters Kluwer), Elsevier, receiving honoraria from Pfizer, receiving research funding to institution from Tesaro, Merck, Seagen, Pfizer, GlaxoSmithKline,

AstraZeneca, Novartis, Genentech/Rosche, Bristol-Myers Squibb/Celgene, and other relationships with Side-Out Foundation, MJH/PER, Curio/Vaniam Group, and Medscape. WDF reports research funding to institution from Celgene, Astellas Pharma, Nerviano Medical Sciences, Pfizer, NovaRX, TRACON Pharma, Biocompatibles, and Propella Therapeutics. SM Swain reports consulting for Genetech/Roche, Daiichi Sankyo, Molecular Templates, AstraZeneca, Aventis Pharma, Jaguar Health, a leadership role at Seagen, receiving travel support from Daiichi Sankyo, Aventis Pharma, Genentech/Roche, and Chugai/Roche, stock or ownership in Seagen, receiving honoraria from Chugai/Roche, and other relationships with Roche, AstraZeneca, and Genentech/Roche. IS, CP, TS, KTS, AC, SS, HCW, NA, SRT, NS, GCN, SKM: Nothing to declare.

## Introduction

Breast cancers can be subclassified based on receptors on tumor cells; these play a critical role in tumor biology and determining the optimal treatment approach for each patient[1]. Hormone (estrogen and/or progesterone) receptor-positive (HR+) and human epidermal growth factor receptor 2 negative (HER2-) breast cancer accounts for approximately 70% of all breast cancers [1]. The cornerstone of treatment for patients with endocrine-sensitive advanced HR+/HER2- breast cancer is endocrine therapy (ET), which consists of selective estrogen receptor modulators (SERM: tamoxifen), aromatase inhibitors (AIs: letrozole, anastrozole, exemestane) or selective estrogen receptor degraders (SERDs: fulvestrant) [2,3]. However, patients often develop resistance to these treatments, and progression of disease. Novel agents have been added to ET with the goal of improving patient outcomes.

When added to ET, the cyclin-dependent kinase 4 and 6 inhibitors (CDK4/6i) palbociclib, ribociclib, and abemaciclib improve progression-free survival (PFS) [4–10]. In the first-line setting, ribociclib also led to a statistically significant improvement in overall survival (OS), when added to an AI [11]. These oral agents have had a profound impact on the outcomes of patients with advanced HR+/HER2- disease, improving cancer-related outcomes while maintaining a good quality of life. CDK4/6i is now the preferred first-line treatment for patients with metastatic HR+/HER2-disease.

Ribociclib is an active drug that is metabolized (into inactive metabolites) by CYP3A [12]. Therefore, the recommended ribociclib dose varies with CYP3A activity. Prior studies identified that ribociclib is metabolized by CYP3A, and concomitant use of strong CYP3A inhibitors increased ribociclib exposure by 3.2-fold [13]. The FDA recommends the use of alternative therapy or a 50% dose reduction (i.e., from 600 mg to 400 mg) if ribociclib is used with strong CYP3A inhibitors [14]. However, it is unknown if dose changes are needed based on variations in baseline CYP3A activity, which is constituted by CYP3A4 and CYP3A5.

Variations in the genes encoding these proteins, *CYP3A4* and *CYP3A5*, could mimic the effect of inhibition of these enzymes. There is currently insufficient evidence regarding the potential influence of *CYP3A4* and *CYP3A5* genotype on ribociclib exposure.[15] For *CYP3A4*, outside of rare variants (e.g., *CYP3A4*22*, *CYP3A4*6*), there is a paucity of evidence describing the broad impacts of *CYP3A4* variants on enzymatic function. In contrast, variations in *CYP3A5* genotype are well established with tacrolimus dosing and pharmacokinetic properties [16]. In brief, patients who express CYP3A5 (i.e., intermediate or normal metabolizers) are more likely to experience subtherapeutic trough levels and require higher doses of tacrolimus. Interestingly, previous evidence, including the FDA label, suggests that African American patients require higher doses of tacrolimus than White patients [16–18]. Although race and genetic ancestry are distinct, these findings align with the fact that patients of African ancestry are likely (~ 85%) to be expressors (normal or intermediate metabolizers) of CYP3A5 [16,17,19]. This is contrary to patients of European ancestry where ~ 85% are CYP3A5 poor metabolizers (CYP3A5 non-expressors) [16] .

CDK4/6i were approved based on large studies. However, there was limited representation of racial and ethnic minorities; for example, in the pivotal trials studying ribociclib, only 41 of 2,066 (< 2%) patients identified as Black [6–8]. This is primarily a concern because the lack of underrepresented patients reflects a potential continuation of disparities in care[20]. Secondly, it also suggests ribociclib dosing was tested in a population that predominantly lacks CYP3A5 expression (poor metabolizers), and other patient populations may require a different dose to achieve similar outcomes in safety and efficacy. Specifically, patients who self-identify as Black or African American were underrepresented in ribociclib studies and there may be differences in ribociclib metabolism and ribociclib exposure based on *CYP3A5* genotype, which could lead to altered clinical outcomes. LEANORA was a prospective study that assessed the pharmacogenomics (PGx) and pharmacokinetics (PK) of ribociclib in self-identified Black patients with advanced breast cancer.

### Results

## Patient population

Between May 2021 and March 2024, 84 patients were reviewed; 63 patients were ineligible (e.g., not metastatic disease, EKG abnormality) or declined to participate, three participants withdrew after providing consent (i.e., participant changed their mind, inability to obtain blood samples for analyses [n = 2]), 3 participants enrolled in the non-Hispanic White (NHW) cohort that closed to due low accrual (**Supplemental figure S1**). Results, including the NHW cohort, are available in **supplemental tables S1-S4**. Fourteen self-identified Black participants with HR+/HER2- advanced breast cancer completed the study, and their data was included in this analysis. Demographic characteristics are described in Table 1. The median age was 61.5 years, and none of the patients identified as Hispanic. The most common sites of metastatic disease were bone, soft tissue, and lung. All patients received ribociclib and ET (letrozole or fulvestrant) per standard of care. The majority (13) of the patients received letrozole, while one received fulvestrant. Three patients received concurrent ovarian suppression.

Table 1
Patient characteristics

| | Participants n (%) N = 14 |
|---|---|
| Median age (range) | 61.5 (54, 66) |
| Race | |
| African American | 14 (100) |
| Ethnicity | |
| Non-Hispanic | 14 (100) |
| Metastatic sites | |
| Bone | 9 (64) |
| Soft tissue | 5 (36) |
| Lung | 3 (21) |
| Adrenal | 1 (7) |
| Liver | 1 (7) |
| Brain | 0 (0) |
| ECOG | |
| 0 | 8 (57) |
| 1 | 6 (43) |
| Smoking History | |
| Active Smoker | 3 (21) |
| Former Smoker | 5 (36) |
| Never Smoker | 6 (43) |
| Endocrine Therapy | |
| Letrozole | 13 (93) |
| Fulvestrant | 1 (7) |

Median and interquartile range are shown for age.

[1]One participant was prescribed two medications that were not recommended (metformin continued through study; atorvastatin held D-1 through D12 after completion of PK sampling) and two participants were prescribed one medication that was not recommended (ondansetron and mirtazapine).

| | Participants n (%) N = 14 |
|---|---|
| Ovarian Suppression Therapy | |
| None | 11 (79) |
| Goserelin or leuprolide | 3 (21) |
| Concomitant Medications | |
| Prohibited medication | 0 (0) |
| Not recommended medication | 3 (21)[1] |
| CYP3A5 Phenotype | |
| Normal Metabolizer (Expressor) | 1 (7) |
| Intermediate Metabolizer (Expressor) | 6 (43) |
| Poor Metabolizer (Non-expressor) | 7 (50) |
| Median and interquartile range are shown for age. | |
| [1]One participant was prescribed two medications that were not recommended (metformin continued through study; atorvastatin held D-1 through D12 after completion of PK sampling) and two participants were prescribed one medication that was not recommended (ondansetron and mirtazapine). | |

## CYP3A5 genotype/phenotype

A total of 50% were CYP3A5 intermediate metabolizers (IM)/ normal metabolizers (NM) (n = 1 NM and n = 6 IM) and 50% (n = 7) poor metabolizers (PM). The genotype was *CYP3A5*1/*1* for the one NM. The IM phenotype included the *CYP3A5*1/*3* (n = 3), *CYP3A5*1/*6* (n = 2), and *CYP3A5*1/*7* (n = 1) genotypes. The PM phenotype included the *CYP3A5*3/*3* (n = 4), *CYP3A5*3/*6* (n = 2), and *CYP3A5*3/*7* (n = 1) genotypes.

## Human Liver Microsome Analysis

Ribociclib metabolism was evaluated in human liver microsomes obtained from three individuals not from this current study, each harboring a different *CYP3A5* genotype: *CYP3A5*1/*1* (NM), *CYP3A5*1/*3* (IM), and *CYP3A5*3/*3* (no CYP3A5 function or PM). Consistent with the importance of CYP3A5 in ribociclib metabolism, the formation of major ribociclib metabolites was correlated with *CYP3A5* genotype status (Fig. 1). Microsomes from a CYP3A5 NM had the greatest ribociclib metabolite formation followed by IM and PM.

## Pharmacokinetic and Pharmacogenetic Findings

The primary endpoint, area under the curve ($AUC_{TAU}$), was similar between CYP3A5 PM (39,230 hr*ng/mL; IQR: 18,745 to 57,566 hr*ng/mL) vs. IM/NM (43,546 hr*ng/mL; IQR: 35,298 to 46,647 hr*ng/mL; p = 0.38). PK properties by CYP3A5 phenotype are summarized in Table 2. Similarly, there were no statistical differences in maximum concentration ($C_{max}$) or in the time to reach $C_{max}$ suggesting that differences in *CYP3A5* genotype did not impact ribociclib exposure.

Table 2
Pharmacokinetics of ribociclib by CYP3A5 phenotype*

| | **CYP3A5 IM/NM** n = 7 | **CYP3A5 PM** n = 7 | **P-value** |
|---|---|---|---|
| $AUC_{tau}$ (hr*ng/mL) | 43,546 (35298, 46647) | 39,230 (18745, 57566) | 0.38 |
| $AUC_{0-6hr}$ (hr*ng/mL) | 14,907 (8254, 15245) | 11,102 (6143, 13651) | 0.26 |
| $C_{max}$ (ng/mL) | 3,140 (1980, 3540) | 3,020 (1300, 3470) | 0.46 |
| $T_{max}$ (hr) | 2.0 (2.0, 4.0) | 3.8 (2.0, 5.9) | 0.48 |

*Median and interquartile range are shown for continuous data.
**Abbreviations:** AUC: area under the curve, $C_{max}$: maximum concentration, IM/NM: intermediate metabolizers/normal metabolizers; PM: poor metabolizers; $T_{max}$: time to reach maximum concentration

## Toxicity profile

Adverse events (AEs) of interest are summarized in Table 3. Overall, the toxicity profile was consistent with previous reports with ribociclib. The most common toxicities were leukopenia, nausea, vomiting, and diarrhea. The reported grade 3 toxicities included neutropenia, nausea, vomiting, transaminitis, and increased creatinine. This study was not powered to assess differences in AEs between PMs and IM/NMs. There was a similar number of all grade toxicities by CYP3A5 phenotype: 100% (7 of 7) vs. 71% (5 of 7) for IM/NM vs. PMs, respectively (p = 0.46). PMs also had a similar number of grade 3 + AEs (29%, 2/7) compared to IM/NMs (29%, 2/7) (p = 1). Table 4 shows a similar change in laboratory values (i.e., transaminases, absolute neutrophil count [ANC]) and QTc by CYP3A5 phenotype between baseline and mid-cycle and end of cycle 1. **Supplemental table S3** provides data about the toxicity profile in the entire study, and **supplemental table S4** provides additional AE data related to QTc prolongation, including one case from cohort 2 where a participant experienced grade 1 QTc prolongation and was unknowingly prescribed a prohibited medication that could prolong the QTc (venlafaxine). Univariate analyses of clinical factors did not identify significant associations with ribociclib AUC (Table 5). These findings should be considered with caution due to the small sample size and because the study was not powered to assess these differences.

Table 3
Adverse events of interest by CYP3A5 phenotype

| | **CYP3A5 IM/NM n = 7** | | **CYP3A5 PM n = 7** | |
|---|---|---|---|---|
| | **Toxicity (any grade)** | **Toxicity (grade 3+)** | **Toxicity (any grade)** | **Toxicity (grade 3+)** |
| **Any AE** | 7 | 2 | 5 | 2 |
| **Hematologic** | | | | |
| Neutropenia | 0 | 0 | 3 | 1 |
| Thrombocytopenia | 1 | 0 | 0 | 0 |
| Leukopenia | 3 | 1 | 2 | 1 |
| Anemia | 2 | 1 | 0 | 0 |
| **Gastrointestinal** | | | | |
| Diarrhea | 1 | 0 | 2 | 0 |
| Nausea | 3 | 1 | 2 | 1 |
| Vomiting | 2 | 1 | 2 | 1 |
| Transaminitis | 1 | 0 | 1 | 1 |
| Constipation | 1 | 0 | 1 | 0 |
| Abdominal pain | 0 | 0 | 1 | 0 |
| Melena | 1 | 0 | 0 | 0 |
| Mucositis | 1 | 0 | 0 | 0 |
| **Cardiac** | | | | |
| QTc Prolongation | 3 | 0 | 1 | 0 |
| Palpitations | 1 | 0 | 0 | 0 |
| Sinus tachycardia | 1 | 0 | 0 | 0 |
| **Metabolism and Nutrition Disorders** | | | | |
| Arthralgia | 1 | 0 | 1 | 0 |

The list of specific types of AEs has been abbreviated. A complete list of recorded adverse events are available in supplemental table S3.

**Abbreviations**: AE: adverse events; IM/NM: intermediate metabolizers/normal metabolizers; PM: poor metabolizers

| | CYP3A5 IM/NM n = 7 | | CYP3A5 PM n = 7 | |
|---|---|---|---|---|
| | Toxicity (any grade) | Toxicity (grade 3+) | Toxicity (any grade) | Toxicity (grade 3+) |
| Back pain | 0 | 0 | 1 | 0 |
| Bone pain | 1 | 0 | 0 | 0 |
| The list of specific types of AEs has been abbreviated. A complete list of recorded adverse events are available in supplemental table S3. | | | | |
| **Abbreviations**: AE: adverse events; IM/NM: intermediate metabolizers/normal metabolizers; PM: poor metabolizers | | | | |

Table 4
Association between CYP3A5 phenotype and laboratory or electrocardiogram values

| Lab Test | Difference at mid-cycle of C1 | | SD | p value | Difference at end of C1 | | SD | p value |
|---|---|---|---|---|---|---|---|---|
| | CYP3A5 IM/PM | CYP3A5 NM | | | CYP3A5 IM/PM | CYP3A5 NM | | |
| ALT (U/L) | -1 (-8, 2) | -4 (-9, 4) | -0.21 | 0.78 | -7 (-10, 3) | -5 (-9, 6) | -0.36 | 0.56 |
| AST (U/L) | 0 (-5, 2) | -2 (-6, 3) | 0.54 | 0.71 | -2 (-6, 5) | -2 (-6, 13) | 0.51 | 0.88 |
| QTc (ms) | 9 (-5, 20) | 18 (-2, 46) | 0.07 | 0.54 | 3 (-1, 28) | 16 (-21, 27) | -0.45 | 0.80 |
| ANC (1000/$mm^3$) | -2 (-2, 0) | -3 (-4, -1) | -1.1 | 0.09 | -2 (-2, -1) | -3 (-6, -2) | -1.1 | 0.10 |
| Abbreviations: ALT: alanine aminotransferase; ANC: absolute neutrophil count; AST: aspartate aminotransferase, C1: cycle 1; IM/NM: intermediate metabolizers/normal metabolizers; PM: poor metabolizers; SD: standard deviation | | | | | | | | |
| Median and interquartile range are shown for continuous data. | | | | | | | | |
| SD represents the standardized difference in CYP3A5 poor metabolizers minus the CYP3A5 IM/PM for the change in each endpoint (post minus baseline). | | | | | | | | |

Table 5
Univariate analysis of clinical factors on ribociclib AUC

| | Level | n | Statistics | p-value[a] |
|---|---|---|---|---|
| | | | correlation[a] | |
| age (years) | - | 14 | 0.43 | 0.1258 |
| weight (kg) | - | 14 | -0.28 | 0.3253 |
| body surface area | - | 14 | -0.34 | 0.2360 |
| creatinine clearance (mL/min) | - | 14 | -0.25 | 0.3825 |
| days of ribociclib therapy | - | 14 | -0.03 | 0.9153 |
| | | | median | |
| ECOG performance status[b] | 0 | 8 | 39200 | 0.5728[c] |
| | 1 | 6 | 43430 | |
| smoking | never | 6 | 41155 | 0.4867[d] |
| | former | 5 | 32798 | |
| | current | 3 | 46647 | |
| | never/former | 11 | 39230 | 0.0879[c] |
| | current | 3 | 46647 | |
| concomitant unrecommended medication[e] | 0 | 11 | 43081 | 0.6612[d] |
| | 1 | 2 | 35679 | |
| | 2 | 1 | 57566 | |

[a] Spearman’s correlation and the corresponding p-value

[b] 0: fully functional, 1: limited functional

[c] an exact Wilcoxon rank sum test-based p-value

[d] an exact Jonckheere-Terpstra trend test-based p-value

[e] the number of unrecommended medications taken by each participant. The two participants taking one unrecommended medication were prescribed a medication that may prolong QT interval (mirtazapine, ondansetron). The participant with two unrecommended medications was prescribed metformin (ribociclib may increase metformin levels) throughout the study and atorvastatin (CYP3A substrate) was paused the day before ribociclib initiation until D13 after completion of sample collection for PK analysis.

**Abbreviations**: AUC: area under the curve

## Exploratory candidate gene analysis

Genetic data were available for all 14 participants in the exploratory candidate gene analysis. Out of 495 variants assessed, 285 variants were identical among all participants. No further evaluation was conducted among those 285 variants. The remaining 210 variants yielded two different genotypes in 106 variants and three different genotypes in 104 variants. This exploratory analysis of 14 participants identified one variant with a $p < 0.05$ for ribociclib $AUC_{TAU}$: *CYP3A4* rs2246709 ($p = 0.0041$), with A/G being associated with elevated $AUC_{TAU}$ compared to A/A (Fig. 2A and 2B).

## Discussion

In the LEANORA trial, we found no association between *CYP3A5* genotype and ribociclib exposure or adverse events among self-identified Black participants. Our findings do not suggest that *CYP3A5* screening is warranted to determine the safety of ribociclib. However, correlative studies of larger trials using ribociclib may provide more insight into interindividual exposure and response to ribociclib. This was seen in the microsome data in which metabolites from a CYP3A5 NM had the greatest ribociclib metabolite formation (inactive) followed by IM and PM, respectively.

These findings are aligned with prior population pharmacokinetics (popPK) and pharmacokinetic/pharmacodynamic (PK/PD) models that found ribociclib pharmacokinetics are not affected by sex or ECOG performance status [21]. We identified weak associations between age and increased ribociclib $AUC_{TAU}$ and weight and decreased $AUC_{TAU}$. This small inverse correlation between body weight and ribociclib exposure is aligned with data included in the FDA review of ribociclib but is not expected to be clinically relevant [22]. Similar to ribociclib, palbociclib is predominantly metabolized by CYP3A [15]. A case report identified that CYP3A5 expression (*CYP3A5*1/*3*, IM) was associated with lower ribociclib plasma concentration [23]. James *et. al.* conducted several studies that identified ~ 63% of ribociclib metabolism is produced by CYP3A [24]. There is significant overlap in the substrates of CYP3A4 and CYP3A5, and it is possible that both impact drug metabolism [13,24]. However, this current study's findings in participants with cancer suggest *CYP3A5* genotype has a minimal role. The recombinant human enzyme studies suggest CYP3A4 is the primary enzyme responsible for ribociclib metabolism [24].

It is possible that genetic variations in *CYP3A4* may be associated with ribociclib pharmacokinetics, but variations in *CYP3A4* are less common. One pre-identified *CYP3A4* variant of interest, *CYP3A4*22*, was present in one participant in this trial, which limited further investigation of that specific variant. Additionally, the exploratory candidate gene analysis identified one variant of *CYP3A4*, rs2246709, potentially associated with ribociclib $AUC_{TAU}$, which happened to be located in *CYP3A4*. However, this is an intronic variant with no obvious impact on enzyme expression or function. A query in the National Institutes of Health (NIH) LDLink did not identify linkage disequilibrium with other variants (highest $r^2$

was 0.36).[25] Future steps could include exploring the impact of rare variants on ribociclib exposure in larger samples or may also explore genetic associations (e.g., *KCNH2*, *SCN5A*, *SNTA1*) with QTc prolongation secondary to ribociclib therapy [26].

This study was focused on identifying a potential association between *CYP3A5* genotype and ribociclib PKs. Therefore, concomitant medications were strictly regulated. In the one case where a patient was unknowingly prescribed a prohibited medication (venlafaxine), administration of this agent was associated with an adverse event (Grade 1 QTc prolongation). The ET was limited to letrozole, fulvestrant with or without ovarian function suppression, given potential interactions of other agents, such as tamoxifen and anastrozole, which could have impacted the PK studies [27].

In terms of ribociclib dosing, the current recommended dose for patients with mBC is 600 mg daily for 21 days, followed by 7-day off treatment. There are prespecified dose reductions based on tolerance. An analysis of the MONALEESA 2, 3, and 7 studies revealed that 42% of patients required a dose reduction, which did not affect the efficacy of the drug [28]. The AMALEE (NCT03822468) study compared the standard 600 mg dose of ribociclib to 400 mg with the same schedule; after a median follow-up of 14 months, the study did not meet its primary endpoint of non-inferiority for the low dose based on objective response rate [29]. However, the study showed a favorable toxicity profile of the lower dose, particularly for neutropenia and QTc prolongation. The approval of cancer-directed therapies has traditionally been based on the maximum tolerated dose, and in recent years, there has been a shift to consider optimal biological doses. The latter is not based on toxicities but rather on other cancer-specific endpoints such as AMALEE [30]. We encourage researchers to rethink study endpoints to improve patient outcomes while limiting toxicity when possible.

Although no difference was found in ribociclib pharmacokinetics by *CYP3A5* genotype, this trial has important implications for clinical practice. It is reassuring for patients and clinicians that the standard of care is not affected by the *CYP3A5* genotype. Additionally, this trial represents the largest known cohort of ribociclib PK data in patients who self-identify as Black.

It is critical to understand the differences between race and ancestry when conducting and interpreting research. Race and ethnicity are social constructs that can be self-ascribed and based on shared physical or social qualities [31]. They do not necessarily reflect genetic ancestry; therefore, using race or ethnicity as proxies for generic ancestry can be inaccurate [19]. Genetic ancestry refers to people in the past to whom an individual is biologically connected [32,33]. Genetic ancestry and genealogy can influence frequencies of genetic variants (e.g., carrier of *HLA-B*15:02* or *CYP3A5*1*). Providing clinical recommendations based on genetic testing in pharmacology remains controversial as the impact of testing on clinical outcomes remains unknown [33–35]. It is likely that race, ethnicity, and genetic ancestry all contribute to patient outcomes. This trial studied an underrepresented population based on race as well as genetic variants more prevalent among those of African ancestry.

Strengths of this trial include that it was a PK and PGx study conducted in participants with cancer and to our knowledge, this is the largest cohort with ribociclib pharmacokinetic data in Black participants. Lastly, the robust sample collection (prior to the ribociclib dose, and 0.5hr, 1hr, 2hr, 4hr, and 6hr) allows for calculation of AUC over a 24-hour period given the reported $T_{max}$ is one to four hours per the FDA label [14].

Limitations of this study include limited sample size for secondary analyses (e.g., adverse events) and for the investigation of rare variants on ribociclib pharmacokinetics. Secondly, it only followed participants through the first cycle, data on long term toxicities and the impact of dose reductions in PKs are not available.

In conclusion, we found no association between *CYP3A5* genotype and ribociclib exposure or adverse events through cycle 1. Ensuring diverse patient representation in clinical trials is critical results that are applicable to the population, so we can continue to improve outcomes of all patients.

### Methods

## Study Design

LEANORA (NCT04657679) was a prospective, observational, multicenter cohort study that assessed the pharmacokinetics and pharmacogenomics of ribociclib. The trial was opened in four sites in the United States (Georgetown Lombardi Comprehensive Cancer Center, Washington DC; MedStar Washington Hospital Center, Washington DC; Tufts Medical Center, Boston, MA; MedStar Franklin Square, Baltimore MD). All participants were followed for the first cycle and prescribed ribociclib 600 mg daily (3 weeks on, 1 week off) plus letrozole 2.5 mg daily or fulvestrant 500 mg on days 1 and 15. Ovarian suppression was required for premenopausal patients, with either goserelin (3.6 mg every 28 days or 10.8 mg every 12 weeks) or leuprolide depot (3.75 mg every 28 days or 11.25 mg every 12 weeks). Dose modifications were provided for ribociclib based on adverse events; however, none of the participants had dose reductions prior to the PGx and PK studies. The institutional review boards (IRBs) approved the study at Georgetown University -and affiliated sites- and Tufts Medical Center. Written informed consent was obtained from all participants. All procedures were in accordance with the ethical standards of the Declaration of Helsinki.

## Patient population

Eligible patients included women with previously untreated HR+/HER2- metastatic or locally advanced breast cancer who self-identified as NHW or African American/Black. Documentation of estrogen receptor (ER) positive and/or progesterone receptor (PR) positive tumor (≥ 1% positive stained cells) based on the most recent tumor biopsy utilizing an assay consistent with local standards. HER2 negative was defined by the American Society of Clinical Oncology/College of American Pathologists (ASCO/CAP) guidelines [36]. Patients were excluded if they were receiving other ET (given for concern for potential drug interaction or increased risk for toxicity) or if they were receiving concurrent medications that could impact CYP3A, other proteins related to ribociclib pharmacokinetics, or risk of adverse events (**Supplemental tables S5-S6**).

Participants were considered postmenopausal if: i) they have had prior bilateral oophorectomy; ii) age ≥ 60 years; iii) age < 60 years and have had amenorrhea for 12 or more months (in the absence of chemotherapy, tamoxifen, toremifene, or ovarian suppression) and follicle-stimulating hormone (FSH) and estradiol in the postmenopausal range per local normal ranges.

The study initially enrolled participants into two independently powered cohorts based on self-identified race: cohort 1) African American or Black participants and cohort 2) NHW participants. However, the planned cohort 2 only enrolled three patients, and due to difficulty enrolling patients, this cohort was closed. In this manuscript, we present the data about cohort 1. However, the results of the patients enrolled in cohort 2 are described in the supplemental material.

## Human Liver Microsome Analysis

Human Liver Microsomes (20 pmol/mL CYP/reaction; 37°C, Seksui Xenotech, Kansas City, KS) were added to preincubated reaction buffer containing 0.5M potassium phosphate buffer (pH 7.4), NADPH solutions A and B according to the manufacturer's instructions (Corning, Corning NY), and ribociclib (Selleckchem, Houston, TX) or tacrolimus (MedChemExpress, Monmouth Junction, NJ) dissolved in acetonitrile at a final concentration of 25μm (0.1% acetonitrile). Aliquots (100 μL) were removed from the reaction immediately after the addition of HLMs (0min) and 60 minutes later. Reactions were terminated by the addition of 100 μL of ice-cold acetonitrile followed by 1 minute of vortexing. Tubes were then centrifuged at maximum speed for 10 minutes at 4°C and harvested supernatant was stored (-20°C) until pharmacokinetic analysis. Bioanalytical measurements of ribociclib and metabolite concentrations were made using an LC-MS/MS assay described in the next section.

## Pharmacokinetic and Pharmacogenetic Testing

Serial blood samples were collected for PK and PGx analyses (Fig. 3). The FDA label reports that ribociclib has an elimination half-life of 32 hours and a maximum plasma concentration ($C_{max}$) of 1 to 4 hours [14]. For sampling to occur at steady-state concentrations, the collection day was scheduled between days 8 and 16 of therapy during cycle 1. Blood samples were collected immediately prior to the ribociclib dose, and 0.5hr ± 5min, 1hr ± 5min, 2hr ± 15min, 4hr ± 15min, and 6hr ± 15min after the daily dose of ribociclib, processed to plasma and stored at -80 C until bioanalysis.

For in vitro metabolism experiments and clinical PK plasma samples, ribociclib total concentrations (i.e. protein bound + unbound) were measured using a validated and robust LC-MS/MS assay using palbociclib as an internal standard. Briefly, samples were mixed with acetonitrile containing palbociclib as an internal standard and run alongside calibration and quality control (QC) standards, ranging from 0.25–50 μM. The analyte (ribociclib) and internal standard were chromatographically separated using a Phenomenex Polar Omega C18 column (5 μm, 2.1x100 mm) on a Shimadzu Prominence HPLC system (Shimadzu, Columbia, MD). A gradient mobile phase consisting of 0.1% formic acid (aq) and 0.1% formic acid in acetonitrile was flowed at a rate of 0.4 mL/min; the injection volume was 5 µL. Following chromatographic separation and elution, the compounds were detected using tandem mass spectrometric detection in the positive ion mode. MRM transitions were m/z 435.3 ◊ 322.2 and m/z 448.4 ◊ 380.2 for ribociclib and palbociclib, respectively. Additionally, ribociclib metabolites were also detection and quantitated based off the parent ribociclib calibration: m/z 421.3 ◊ 322.2 (Demethyl); m/z 407.3 ◊ 322.2 (Di-demethyl); m/z 451.3 ◊ 338.3 (Hydroxyl); m/z 449.3 ◊ 336.3 (Oxidation).

A non-compartmental approach to clinical plasma PK analysis was employed using Phoenix WinNonlin v8.3 (Certara Corp, Cary, NC) that was validated per FDA 21CFR Part 11 regulations. The maximum observed plasma concentration ($C_{max}$) and the time of $C_{max}$ ($T_{max}$) were recorded as observed values. The area under the concentration-time curve over the first 6 hours post-dose and over the dosing interval during steady-state ($AUC_{0-6hr}$ and $AUC_{tau}$, respectively) was calculated using the linear-up/log-down trapezoidal method.

Ten milliliters of frozen whole blood was used to isolate DNA via the Wizard® Genomic DNA Purification Kit (Promega) according to manufacturer instructions. PGx data were obtained from the PharmacoScan™ (ThermoFisher) microarray per manufacturer instructions, which tests 4,627 pharmacogenetic markers in 1,191 genes. This test included the three variants known to correspond with no function (i.e., *CYP3A5*3, *6*, and **7*). In accordance with Clinical Pharmacogenetics Consortium (CPIC) guidelines, Phenotypes assigned: poor metabolizers (PM, 2 variant alleles), intermediate metabolism (IM; 1 variant allele), NM (0 variant alleles) [16].

## Endpoints

The primary endpoint was ribociclib $AUC_{tau}$ (visit between days 8–16 of cycle 1) CYP3A5 PM and CYP3A5 IM/NM. Secondary endpoints included additional ribociclib pharmacokinetic properties and adverse events (AEs). Pharmacokinetic properties were maximum concentration ($C_{max}$), $AUC_{0-6hr}$, the time to reach Cmax ($T_{max}$), clearance, volume of distribution($v_d$), and elimination half-life. Secondary safety endpoints included change in QTc interval and occurrence of neutropenia or aspartate aminotransferase (AST) or alanine aminotransferase (ALT) elevations per Common Terminology Criteria for Adverse Events (CTCAE) version 5.0. An appearance or worsening of an undesirable sign, symptom, or medical condition after initiation of ribociclib per the CTCAE was considered an adverse event regardless of attribution to ribociclib.

## Sample Size Calculation

Sample size accounted for *CYP3A5* allelic frequencies for *CYP3A5* from CPIC and PharmGKB, which were translated to phenotypes via the Hardy-Weinberg Eq. 1[6]. Based on known frequencies for African Americans (normal metabolism: 35%; intermediate metabolism: 48%; poor metabolism: 16%), a sample size of 18 would provide 80% power at an $\alpha = 0.05$ to detect a 2-fold change in AUC (SD is 0.56) between CYP3A5 PM vs. CYP3A5 expressors (i.e., CYP3A5 IM/NM). After the enrollment of 14 participants who self-identified as African American or Black, the distribution of CYP3A5 results was a 1:1 split for

CYP3A5 IM/NM vs. PM. A second sample size calculation identified this cohort would provide 86.5% power to detect a 2 vs 1 difference in AUC pending the SD is 0.56. Alpha set at 0.05. The cohort that enrolled self-identified non-Hispanic White participants was terminated prematurely due to low accrual (n = 3). A buccal swab for *CYP3A5* genotyping (Kailos Genetics) was obtained during the screening period for each patient to ensure adequate sample sizes of each phenotype per cohort.

## Statistical analysis

The area under the curve ($AUC_{tau}$) between CYP3A5 PMs vs. IM/NMs was compared with the exact Wilcoxon rank-sum test due to small sample sizes, with alpha set to 0.05. Secondly, a multiple regression analysis was intended to identify variables associated with ribociclib AUC. Covariates to be assessed include CYP3A5 metabolism status, CYP3A4 metabolism status, renal function, liver function, age, race, weight, sex, use of “medication not recommended”, AI, number of ribociclib doses taken. However, the multiple linear regression was not conducted due to the inability to provide valid inference with the small sample size. Fisher’s exact test assessed the AEs and grade 3 + AEs to day 28. Descriptive statistics were used to characterize the data profile, frequency, and percentages for categorical variables and mean (SD) or median [IQR] for continuous variables based on the data normality. Laboratory and electrocardiographic assessments were done per the current standard of care at baseline, close to C1D15, and at the end of the first cycle [14]. The QT interval was corrected using the Bazett method. Analysis was performed using SAS™ software (v 9.1; Cary, NC, USA) and R Statistical Software (v4.3.1; R Core Team 2023).

## Exploratory Candidate Gene Analysis

We conducted an exploratory candidate gene analysis among measured variants in genes that were previously linked to ribociclib pharmacokinetics. The objective of this analysis was to assess if there was a strong genetic effect on ribociclib $AUC_{TAU}$. A literature review in May 2024 (search terms “ribociclib AND pharmacokinetics” in PubMed) identified 80 articles. Articles were screened for content related to genes or proteins related to ribociclib metabolism. Seventeen articles met inclusion and led to the identification of 15 genes of interest: *ABCB1, ABCG2, CYP1A2, CYP2C19, CYP2C9, CYP3A4, CYP3A5, FMO1, FMO3, NR1/2, PPARα, POR, SLCO1B1, SLCO1B3, SULT2A1*. Genetic data were available for 14 of 15 genes, with it unavailable for *PPARα*. The list of variants interrogated is available in **supplemental table S7.** The association of each of the variants with ribociclib $AUC_{TAU}$ was evaluated by an exact Wilcoxon rank sum test or a Kruskal-Wallis test if there are two or three types of genotype within each variant, respectively. All the statistical results should be interpreted as exploratory and descriptive with two-tailed p-values unadjusted for multiple comparisons.

### Abbreviations

AUC

area under the curve, $C_{max}$:maximum concentration, IM/NM:intermediate metabolizers/normal metabolizers

PM

poor metabolizers

$T_{max}$

time to reach maximum concentration

## Declarations

**Data availability:** The data set generated and analyzed during the LEANORA study are available upon request to the corresponding author (sms248@georgetown.edu)

**Acknowledgments:** The authors thank Dr. Asma Dilawari for her help with study design and operational considerations, Hiwot Guebre-Xabiher, Terry Jeffs, Ramamohana Jonnala, Meghan Mavredes, Dionyssia Clagett, Ryan P. Brown, and Jude Uwah for operational support, Ricki Fairley for patient advocacy input. We thank all of the patients who participated in the trial and their caregivers.

**Author contributions:** IS and DMS contributed equally as co-first authors. Conception and Design: IS, DMS, WDF, SMS. Provision of study materials or patients: IS, AC, CG, NA, KW, CM, ST, and CI. Collection and assembly of data: IS, DMS, NS, and SMS. Data analysis and interpretation: IS, DMA, CP, TS, KTS, MT, SS, HCW, GN, WDF, and SMS Manuscript writing and final approval of manuscript: All authors. Accountable for all aspects of work all authors.

**COI:** DMS reports research funding to institution from Kailos Genetics, Inc. MTT reports receiving honoria from AstraZeneca, Incyce, Otsuka, Sanofi Pasteur, consulting for American Gene Technologies, and receiving research funding to institution from Genentech. NHB reports they or an immediate family member have consulted for Catena, a leadership role for Seagen, stock or ownership in Seagen, Lilly, Gilead Sciences, and Pfizer. CG reports consulting for Daiichi Sankyo, Illy, Biotheranostics, and Pfizer and Speakers' Bureau for Daiichi Sankyo/UCB Japan. KDW reports consults for Biotheranostics and receiving honoraria from MHJ Life Sciences. CBM reports receiving research funding to institution from Pfizer and Cantex Pharmaceuticals. CI reports consulting for Pfizer, Novartis, Puma Biotechnology, Seagen, Ion Solutions, AstraZeneca/MedImmune, Gilead Sciences, receiving travel support from Pfizer, holding patents, royalties, or other intellectual property from McGraw Hill Publishing, UpToDate (Wolters Kluwer), Elsevier, receiving honoraria from Pfizer, receiving research funding to institution from Tesaro, Merck, Seagen, Pfizer, GlaxoSmithKline, AstraZeneca, Novartis, Genentech/Rosche, Bristol-Myers Squibb/Celgene, and other relationships with Side-Out Foundation, MJH/PER, Curio/Vaniam Group, and Medscape. WDF reports research funding to institution from Celgene, Astellas Pharma, Nerviano Medical Sciences, Pfizer, NovaRX, TRACON Pharma, Biocompatibles, and Propella Therapeutics. SM Swain reports consulting for Genetech/Roche, Daiichi Sankyo, Molecular Templates, AstraZeneca, Aventis Pharma, Jaguar Health, a leadership role at Seagen, receiving travel support from Daiichi Sankyo, Aventis Pharma, Genentech/Roche, and Chugai/Roche, stock or ownership in Seagen, receiving honoraria from Chugai/Roche, and other relationships with Roche, AstraZeneca, and Genentech/Roche. IS, CP, TS, KTS, AC, SS, HCW, NA, SRT, NS, GCN, SKM: Nothing to declare.

**Funding:** This project has been funded and/or supported in whole or in part with BCRF-20-156, Conquer Cancer-ASCO Young Investigator Award, Georgetown Lombardi Comprehensive Cancer Center - Core grant (P30CA051008), Grant # UL1TR000101 (previously UL1RR031975) from the National Center for Advancing Translational Sciences (NCATS), National Institutes of Health (NIH), through the Clinical and Translational ScienceAwards Program (CTSA), a trademark of DHHS, part of the Roadmap Initiative, "Re-Engineering the Clinical Research Enterprise.

## Figures

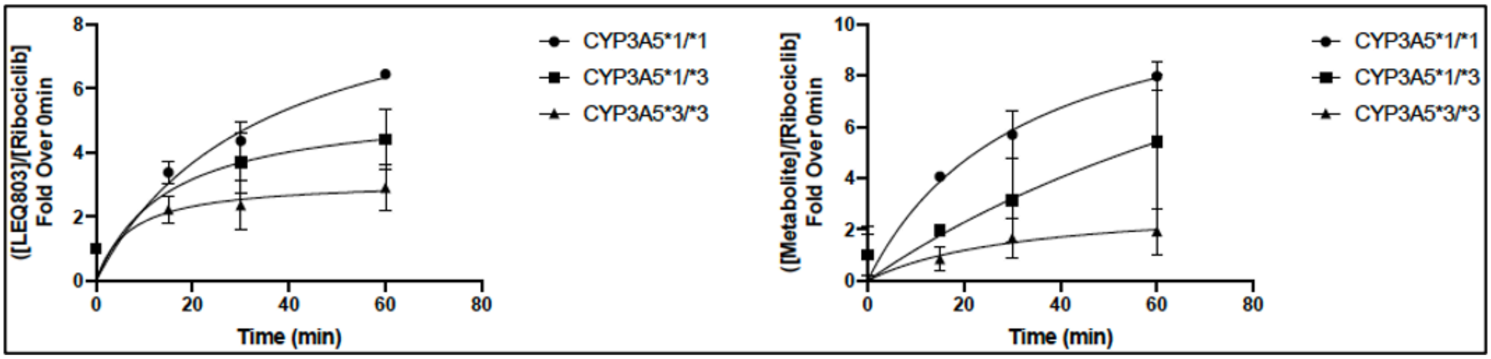

**Figure 1**

Effect of *CYP3A5*genotype on ribociclib metabolism in human liver microsomes
Formation of major ribociclib metabolites over time was correlated with *CYP3A5*genotype status. Human liver microsomes from a CYP3A5 NM (*CYP3A5 *1/*1*) had the greatest ribociclib metabolite formation followed by IM (*CYP3A5 *1/*3*) and PM (*CYP3A5 *3/*3*). Graphs represent 3 technical replicates per datapoint, error bars show standard deviation.

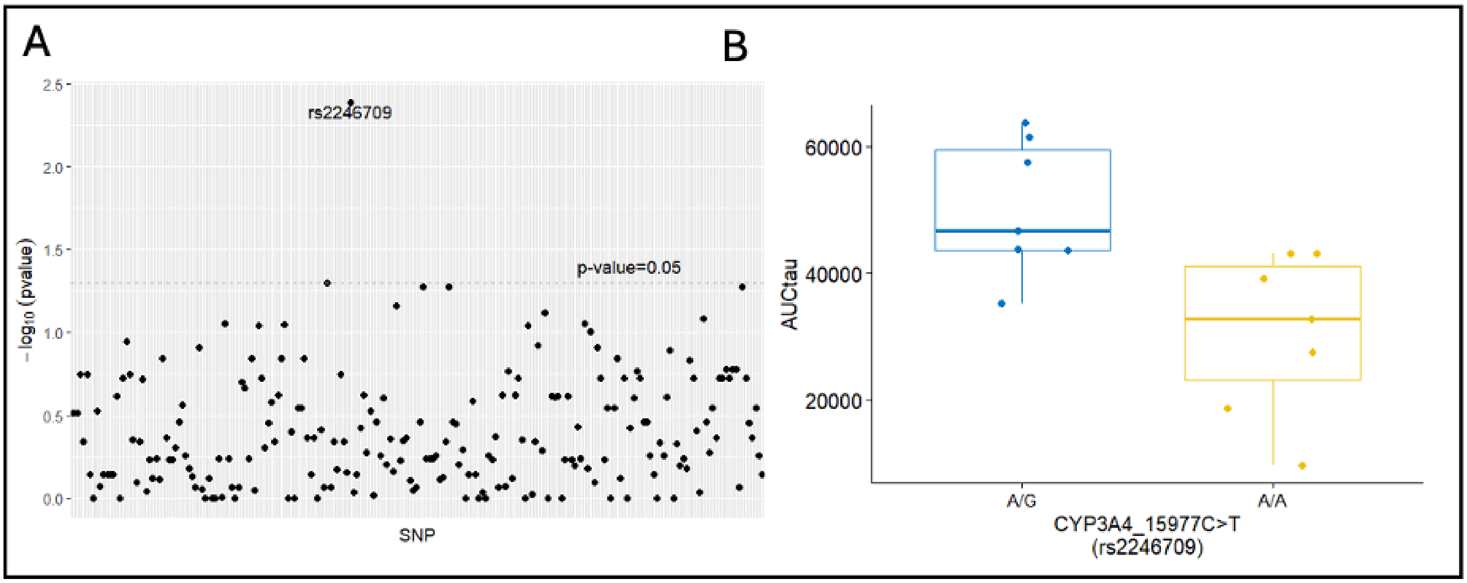

**Figure 2**

P-values by different SNPs of interest and AUCtau by rs2246709

**2A** Genetic data were available for all 14 exploratory candidate gene analysis participants. Variants are plotted by p-value with a dotted line representing a p-value of 0.05. This exploratory analysis of 14 participants identified one variant with a $p < 0.05$ for ribociclib AUCTAU: CYP3A4 rs2246709 ($p = 0.0041$).
**2B.** This exploratory analysis identified an association between ribociclib area under the curve (AUC) in participants with a *CYP3A4* variant (rs2246709), showing a higher AUC in those with A/G relative to those with A/A (p=0.0041).

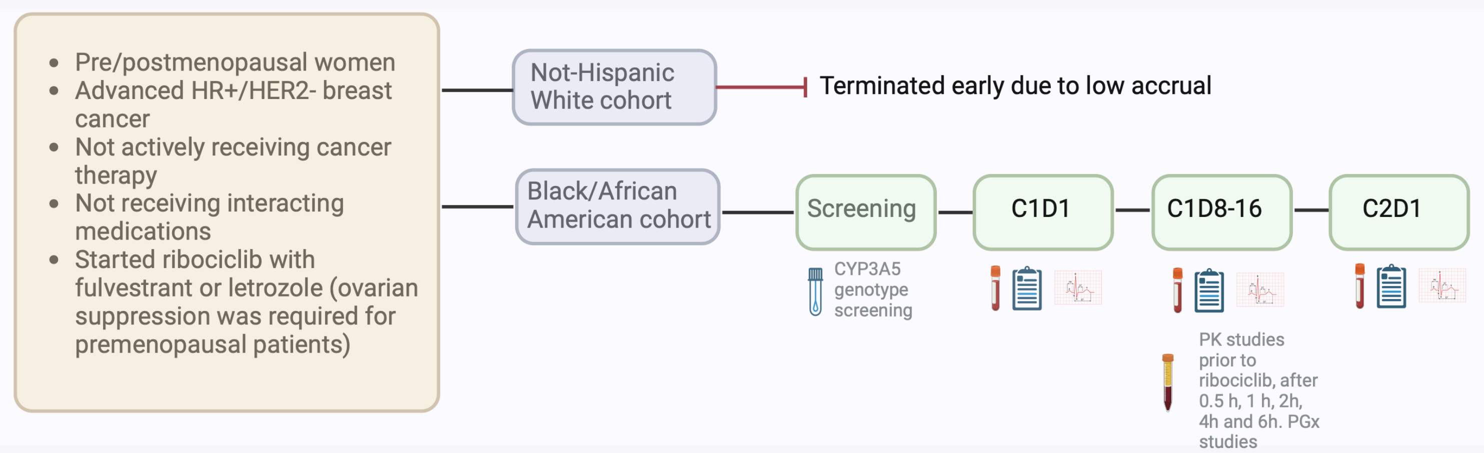

**Figure 3**

Study design

During screening, a swab *CYP3A5* genotype was obtained to ensure adequate power. On C1D1, C1D8-16, and C2D1, blood counts and chemistries were obtained, as well as an electrocardiogram per standard of care. The list of concurrent medications was also obtained, and patients received a drug diary and patient-reported outcomes (CTCAE-PRO) questionnaires. Created with BioRender.com

## Supplementary Files

This is a list of supplementary files associated with this preprint. Click to download.

- LEANORA7.3supplemental.pdf
